# Anti-Yo Associated Paraneoplastic Cerebellar Degeneration in a Man with Large Cell Cancer of the Lung

**DOI:** 10.1155/2013/725936

**Published:** 2013-09-19

**Authors:** Linda Hasadsri, James Lee, Bonnie H. Wang, Lalitha Yekkirala, Mingtao Wang

**Affiliations:** ^1^Department of Laboratory Medicine and Pathology, Mayo Clinic, 200 First Street SW, Rochester, MN 55905, USA; ^2^Department of Internal Medicine, University of Illinois Urbana-Champaign, 611 W. Park Street, Urbana, IL 61801, USA; ^3^Department of Neurology, University of Illinois Urbana-Champaign, 611 W. Park Street, Urbana, IL 61801, USA

## Abstract

Purkinje cell cytoplasmic antibody type 1 (PCA-1), or anti-Yo, is the most frequently detected autoantibody in paraneoplastic cerebellar degeneration (PCD). The vast majority of cases of anti-Yo PCD, however, occur in females over 60 years old and are associated with gynecologic tumors. Only 10 cases have been reported in males, and only 2 were associated with cancer of the lung. Here we describe the youngest known case of PCA-1 positive PCD in a male, whose lung tumor was undetectable even on FDG-PET.

## 1. Introduction

Paraneoplastic neurological syndromes (PNS) are rare disorders of the central and/or peripheral nervous system due to the remote effects of, rather than directly caused by, an underlying malignancy or its metastases. They occur in less than 1% of patients with cancer [1], though paraneoplastic peripheral neuropathy can occur in 50% of patients with osteosclerotic myeloma [2], myasthenia gravis in 10–15% of patients with thymoma [3], and Lambert-Eaton myasthenic syndrome in up to 3% of patients with neoplasms of the lung [4]. The remaining PNS are so uncommon that their exact incidence has not been established [5]. Of these conditions, paraneoplastic cerebellar degeneration (PCD), also known as subacute cerebellar ataxia, is the most common paraneoplastic disease of the brain [6]. It is characterized by severe pancerebellar dysfunction, typically beginning with gait ataxia and progressing, over weeks to months, to severe, symmetrical truncal and limb ataxia, often with dysarthria and nystagmus [7, 8]. Pathological hallmarks of PCD include widespread loss of Purkinje cells and the presence of highly specific antineuronal antibodies in the serum and cerebrospinal fluid (CSF) [9]. Less than 250 cases have been documented in the literature [10], however, and only half of these patients have had positive serological markers for PCD [7].

Purkinje cell cytoplasmic antibody type 1 (PCA-1), or anti-Yo, is the most frequently detected autoantibody in subacute cerebellar degeneration, followed by anti-Hu, anti-Tr, anti-Ri, and anti-mGluR1 [11]. The vast majority of cases of PCD associated with anti-Yo, however, occur in females over the age of 60 years old and are associated with tumors of the ovary, uterus, and breast [8, 12, 13]. Only 10 cases have been reported in males, of which only 2 were associated with cancer of the lung [12–22]. Here we describe the youngest known case of PCA-1 positive PCD in a male, whose tumor was undetected even on FDG-PET. Large cell adenocarcinoma of the lung was revealed on autopsy, making this the third case of non-small cell lung cancer associated with anti-Yo PCD.

## 2. Case Presentation

A 49-year-old white male was initially admitted for a 2-week history of progressive vertigo, ataxia, and slurred speech. He also complained of one episode of nausea and vomiting on the day prior to admission, and due to his disequilibrium he had fallen 3-4 times. He denied any fever, headache, syncope, diplopia, or changes in hearing. His coexisting conditions included seizure disorder of unknown etiology with no history of intractable seizures and with his last seizure having occurred over a decade ago; bipolar disorder; chronic lower back pain secondary to mechanical injury, chronic obstructive pulmonary disease (COPD); marijuana abuse and a 32 pack-year history of smoking. He denied any history of excessive alcohol intake. Four years prior to admission, he underwent surgical removal of a suspicious mass in the upper lobe of his left lung, which pathology later revealed to be a benign, necrotizing granuloma. Neurological examination revealed mild dysarthria with intact language and cognition, significant horizontal nystagmus bilaterally, dysdiadochokinesia, and dysmetria. The patient was unable to walk on his own, and significant ataxia was observed on assisted ambulation. No focal weakness or decreased muscle tone was noted. Deep tendon reflexes were 2+ and symmetric with flexor plantar response. Routine laboratory analyses were unremarkable. Blood alcohol levels were within normal limits. CT scan and MRI of the brain with and without contrast revealed no intracranial hemorrhage, ischemic infarction, or mass. There was no abnormal leptomeningeal nor diploic space enhancement. EEG was abnormal with focal slowing activity at the left temporal area with occasional sharp wave activity in the left frontal and parietal regions. CSF examination showed elevated protein (110 mg/dL) and predominantly lymphocytic pleocytosis (23 WBC/mm^3^). CSF gram stain and cultures were negative, as were a bacterial meningitis panel and herpes simplex rapid PCR. Neuron specific enolase, anti-GM1, and anti-GQ1B antibodies were also undetectable. Antinuclear antibodies (ANA) were negative, and a syphilis RPR screen was nonreactive. The patient was started on meclizine and methylprednisolone, and his vertigo resolved. His ataxia also improved somewhat, and he was discharged after 3 days with a possible diagnosis of viral cerebellitis, treated with only supportive care.

Two weeks later, the patient presented to the emergency department with altered mental status and worsening ataxia and dysarthria. He was disheveled, emotionally labile, and inappropriate, vocalizing thoughts of physical violence but without intent, fluctuating between episodes of anger and tearfulness. His speech had become increasingly slurred, and he was unable to stand without assistance. Neurological examination was also significant for bilateral horizontal nystagmus on lateral gaze and decreased muscle strength and pinprick sensation in both the upper and lower extremities. The patient's attention, concentration, and judgment were all poor. Routine hematology and biochemistry were normal. Multiplanar, multiecho MRI of the brain with and without contrast revealed mild generalized cerebral and cerebellar atrophy but no other abnormalities. A diagnosis of paraneoplastic neurological syndrome (PNS) was suspected but a neoencephalitis paraneoplastic panel was negative. No definitive diagnosis could be established, and, at his family's request, the patient was discharged to a nursing home.

Over the next month, the patient experienced a 30 lb weight loss, and his neurological condition continued to deteriorate. He was readmitted after he developed dysphagia and could no longer tolerate liquids or solid food. By this time, the patient was bedridden and oriented only to his name; when given choices, he could correctly identify his location but not the month or year. His speech had become completely unintelligible and hypophonic. He had lateral gaze nystagmus bilaterally, worse on the right side, and mild, diffuse, bilateral weakness along with symmetric hyporeflexia with a weakly flexor plantar response. CSF cytology showed only mature lymphocytes, monocytes, and peripheral blood. A high resolution CT scan of the chest revealed right paratracheal lymphadenopathy and indeterminant nodules in the lung, as well as a spiculated nodule in the right upper lobe measuring 0.8 × 0.7 cm. PET showed no increased FDG uptake within the nodules, however, and fine needle aspiration and bronchial washing of the right upper lobe showed no evidence of malignancy. The patient was transferred to a tertiary referral hospital where an extensive panel of laboratory tests for paraneoplastic antibodies was performed on his cerebrospinal fluid, which subsequently identified Purkinje cell antibody type 1 (anti-Yo). After a lengthy discussion with the patient's family regarding his poor prognosis, the decision was made to not initiate tube feeding or pursue any further intervention. The patient was placed in hospice care, where he died of cachexia 2 weeks later. Postmortem examination revealed a white, spiculated right upper lobe nodule measuring 0.8 cm and puckering the pleural surface above it. Histologic evaluation revealed a large cell, poorly differentiated adenocarcinoma with robust, benign-appearing lymphocytic infiltrate. Histopathologic examination of the cerebellum revealed widespread but incomplete loss of Purkinje cells, with many of the remaining cells showing evidence of eosinophilic necrosis. Accompanying the Purkinje cell loss was widespread Bergmann's gliosis, scattered microglial proliferation within the molecular layer, patchy leptomeningeal and focal perivascular infiltrates of benign-appearing lymphocytes, and white matter gliosis with rare microglial nodules. Microglial nodules were also present within the dentate nucleus with associated neuronophagia.

## 3. Discussion

Paraneoplastic cerebellar degeneration (PCD) is the most common paraneoplastic syndrome affecting the brain, but occurs in less than 1/10,000 patients with cancer [23, 24]. It is characterized by acute to subacute onset of gait ataxia progressing to, over the course of weeks to months, truncal and limb ataxia, with dysarthria and often nystagmus (especially rotary or vertical) and diplopia [7, 25, 26].

Cognitive impairment occurs in 20–50% of patients and primarily involves functions of the frontal and temporal lobes [8, 24, 27]. Autopsy typically reveals, as seen in our patient, a nearly complete loss of cerebellar Purkinje neurons with variable loss of granule cells, as well as astrocyte and microglial proliferation [24]. A mild lymphocytic infiltrate may be seen in the leptomeninges and perivascularly in the deep cerebellar nuclei, but not in the cerebellar cortex. This is in contrast to anti-Hu PCD, in which prominent inflammatory infiltrates are found in the cerebellum and brainstem as well as other locations throughout the brain [28]. At least 12 onconeural antibodies are highly specific for PCD, including anti-Yo (PCA-1), anti-PCA2, anti-Hu, anti-Tr, anti-Ri, anti- mGluR1, anti-Zic4, anti-Ma, anti-CV2/CRMP5, anti-VGCC, and anti-ANNA3 [25]. Anti-Yo, also known as Purkinje cell cytoplasmic antibody type 1 (PCA-1), is the most frequently detected antibody [11], though only 50% of patients with PCD have detectable antibodies in their serum or CSF [27]. Therefore, the absence of such antibodies cannot rule out the diagnosis of PCD.

The pathogenesis of PCD is not fully known, but is thought to be caused by autoimmunity against antigens expressed by both the underlying tumor and normal neural tissue, leading to humoral and cytotoxic T-cell-mediated destruction of neurons [29, 30]. PCA-1 itself is not thought to be pathogenic as its target antigen, human cerebellar degeneration-related protein-2 (cdr2), is intracellular and therefore inaccessible to circulating antibodies [31, 32]. Cdr2 is normally found in only the cytoplasm of brainstem neurons and Purkinje cells [33–35], but is aberrantly expressed in 62% of ovarian tumors despite being absent in normal ovarian tissue [36]. Moreover, expanded populations of cdr2-specific cytotoxic T lymphocytes (CTLs) have been detected in the blood and cerebrospinal fluid of PCD patients, suggesting that a CTL-mediated attack on neural cells may serve as the basis of the irreversible neurological impairment seen in patients with PCA-1 positive PCD [37].

In up to two-thirds of patients with paraneoplastic cerebellar degeneration, the neurologic symptoms precede the diagnosis of cancer [13]. Moreover, approximately 50% of cases of subacute pancerebellar disorder presenting in middle-age are due to PCD, and 67% of affected individuals are subsequently found to have cancer within a few years [23, 38]. Recognition of new-onset cerebellar dysfunction is therefore crucial as it may be the main presenting feature of an occult tumor. In addition to the clinical presentation, oligoclonal bands in the CSF, with or without concomitant pleocytosis, are also a common laboratory finding in paraneoplastic neurological syndromes [39] and are seen in approximately 60% of patients with PCD [7]. Magnetic resonance imaging (MRI), however, is “remarkably unremarkable” in anti-Yo positive PCD, though abnormalities such as cerebellar atrophy and evidence of cerebellar or extracerebellar inflammation can be seen, especially during later stages of the disease [10, 40, 41]. In one study of 19 anti-Yo positive patients, 7 had no detectable malignancy despite mammography, CT, and MRI, but in each case laparotomy revealed a small, undifferentiated carcinoma in the pelvis [42]. Use of positron emission tomography with fluorodeoxyglucose (FDG-PET), on the other hand, was able to detect tumors where none had been detected by classic radiological methods in 3 out of 4 patients with anti-Yo PCD [22]. There have additionally been 2 other reports of patients with high anti-Yo titers in which an occult tumor was discovered by FDG-PET where other imaging modalities had failed to reveal one [43, 44]. However, as false-positive and false-negative results can occur with FDG-PET, it is currently recommended that FDG-PET be reserved for seropositive patients when conventional imaging fails to identify a tumor or when lesions are difficult to biopsy [7, 21, 22].

Over 200 cases of PCD have been reported in the literature, but PCA-1 antibodies are almost exclusively seen in women, usually in carcinomas of the ovary, uterus, and breast [8]. Only one documented patient over the past 22 years has had vertigo as the predominant presentation [32]. Furthermore, only 10 cases of anti-Yo positive PCD have been reported in men, and only 2 thus far have been associated with adenocarcinoma of the lung [12–22]. The present case is remarkable in that the patient was a male with an undetectable lung tumor despite an extensive diagnostic workup, including FDG-PET [21]. In one series of 38 seropositive patients, all of whom were female, only one had no cancer detected even at laparotomy [42]. In another study, only 3 out of 55 anti-Yo positive patients had no detectable underlying malignancy, even at postmortem in one [8]. Our patient was also a smoker, which is much more commonly associated with antibodies against Hu linked to small cell lung carcinoma (SCLC) [15].

Multiple studies have shown that patients with PCA-1 autoimmunity have a far worse neurological prognosis than PCD patients who test positive for other antibody types [32, 45]. Tumor progression is generally the most frequent cause of death in PCD rather than the neurological disease, as patients can survive for years unless dysphagia due to cerebellar degeneration leads to aspiration pneumonia [46]. In one study, however, 67% of deceased anti-Yo patients died of a neurological cause [11]. No standard treatment protocol currently exists for PCD and, with few exceptions, the use of IV immunoglobulins (IVIG), plasmapheresis, cyclophosphamide, and corticosteroids, whether alone or in combination, has not demonstrated long-term efficacy [25]. Slight fluctuations in neurological status are frequent in PCA-1 associated PCD, but a significant improvement rarely if ever occurs in this aggressive disease [11]. Symptomatic treatment of cerebellar signs could include neurorehabilitation with swallowing and speech therapy, and modest additional gains may be seen with propranolol or antiepileptic drugs [7]. In addition, as autoimmune disorders such as multiple sclerosis are being increasingly treated with hematopoietic stem cell therapy following myelosuppressive conditioning [47, 48], autologous stem cell transplantation might be considered in select patients with an aggressive course of PCD [11].

Ultimately, the prognosis for anti-Yo positive PCD is quite poor, as even chemotherapy or other treatment of the primary tumor rarely results in improved outcome [49, 50]. In one series of 55 anti-Yo positive patients, 22 underwent at least 5 plasma exchanges with a moderate sustained improvement in one and mild transient improvement in the other 4 [8]. Another 17 were treated with high dose corticosteroids, but only one patient had a mild transient improvement while another had moderate improvement with treatment of the underlying tumor [8]. In another series of 16 patients, no benefit resulted from steroids or plasmapheresis, but 2 had a slight improvement upon treatment of the underlying cancer [27]. In an additional study of 3 patients with anti-Yo associated PCD, none demonstrated significant neurological improvement despite attempts at treatment with plasma exchange, IVIG, antineoplastic surgery, cytotoxic chemotherapy, or immunosuppression [24]. A more recent study of 67 patients showed sustained neurological stabilization or mild improvement in only 10 cases (15%), 5 of whom received both chemotherapy and immunotherapy in addition to IVIG; 3 patients received chemotherapy alone, and 2 patients received immunotherapy alone [32]. It should be noted, however, that in most cases of PCD, the neurological syndrome has stabilized by the time treatment is instituted, meaning that permanent Purkinje cell damage and neuronal loss is likely to have already occurred [8]. As such, despite successful treatment of the primary tumor in many cases, the paraneoplastic symptoms often continue to have a more significant negative impact on patients' quality of life than does the underlying disease [51].
